# Effective cultivation of microalgae for biofuel production: a pilot-scale evaluation of a novel oleaginous microalga *Graesiella* sp. WBG-1

**DOI:** 10.1186/s13068-016-0541-y

**Published:** 2016-06-13

**Authors:** Xiaobin Wen, Kui Du, Zhongjie Wang, Xinan Peng, Liming Luo, Huanping Tao, Yan Xu, Dan Zhang, Yahong Geng, Yeguang Li

**Affiliations:** Key Laboratory of Pant Germplasm Enhancement and Specialty Agriculture, Wuhan Botanical Garden, Chinese Academy of Sciences, Wuhan, 430074 China; University of Chinese Academy of Sciences, Beijing, 100049 China; Department of Pathology and Immunology, Baylor College of Medicine, Houston, TX 78703 USA

**Keywords:** *Graesiella*, Microalgae selection, Lipid, Mass culture, Open raceway pond

## Abstract

**Background:**

Commercial production of microalgal biodiesel is not yet economically viable, largely because of low storage lipid yield in microalgae mass cultivation. Selection of lipid-rich microalgae, thus, becomes one of the key research topics for microalgal biodiesel production. However, the laboratory screening protocols alone cannot predict the ability of the strains to dominate and perform in outdoor ponds. Comprehensive assessment of microalgae species should be performed not only under the laboratory conditions, but also in the fields.

**Results:**

Laboratory investigations using a bubbled column photobioreactor indicated the microalga *Graesiella* sp. WBG-1 to be the most productive species among the 63 Chlorophyta strains. In a 10 L reactor, mimicking the industrial circular pond, *Graesiella* sp. WBG-1 produced 12.03 g biomass m^−2^ day^−1^ and 5.44 g lipids (45.23 % DW) m^−2^ day^−1^ under 15 mol m^−2^ day^−1^ artificial light irradiations. The lipid content decreased to ~34 % DW when the microalga was cultured in 30 L tank PBR under natural solar irradiations, but the decline of lipid content with scaling up was the minimum among the tested strains. Based on these results, the microalga was further tested for its lipid production and culture competitiveness using a pilot-scale raceway pond (200 m^2^ illuminated area, culture volume 40,000 L). Consequently, *Graesiella* sp. WBG-1 maintained a high lipid content (33.4 % DW), of which ~90 % was storage TAGs. Results from the outdoor experiments indicated the nice adaptability of the *Graesiella* sp. WBG-1 to strong and fluctuating natural solar irradiance and temperature, and also demonstrated several other features, such as large cell size (easy for harvest and resistant to swallow by protozoa) and tolerance to high culture pH (helpful to CO_2_ fixation).

**Conclusions:**

*Graesiella* sp. WBG-1 was a promising strain capable of accumulating large amount of storage lipid under nature solar irradiance and temperature. The high lipid content of 33.4 % DW was achieved for the first time in pilot-scale raceway pond. The results also provide evidence for the feasibility of using low-cost raceway pond for autotrophic cultivation of microalgae for biodiesel production.

**Electronic supplementary material:**

The online version of this article (doi:10.1186/s13068-016-0541-y) contains supplementary material, which is available to authorized users.

## Background

In recent years, mass production of microalgae has been extensively exploited for applications in the industry of food, aquaculture, and bioenergy, and especially for biodiesel production [1, 2]. However, there is still a large margin to improve economic yield for large-scale biofuel production [3, 4], since fossil fuel is still much cheaper and serves as the dominant energy source nowadays.

One of the obstacles making microalgal biodiesel commercially unfeasible is the low yield of storage lipid in outdoor microalgae mass cultivation [3, 5]. So far, the algal biomass productivities achieved in large-scale raceway pond do not exceed 20–40 g dry weight m^−2^ day^−1^, and a maximum solar-to-biomass conversion efficiency of 3 % was reported [6, 7]. Furthermore, high lipid accumulation is more difficult to achieve. Although *Chlorella*, *Scenedesmus*, *Neochloris,* and *Nanochloropsis* strains are repeatedly reported to give an average lipid content of 40–60 % in dry cell mass in laboratory [8, 9], outdoor cultivation of these strains has only been accomplished on very small scale and a total lipid content of 30 % has rarely been achieved [10–12]. The Aquatic Species Program (ASP) spent considerable effort in isolation, screening, genetic improvement, and outdoor cultivation of microalgae strains. At its peak, the collection of ASP contained over 3000 strains of lipid-rich microalgae [13]. However, there is still lack of robust strains capable of high lipid productivity in outdoor large-scale cultivation [6, 13]. In its close-out report, ASP pointed out that the laboratory-level screening protocols have relatively little predictive power for the ability of the strains to dominate and perform in outdoor ponds [13].

To fill the gap between laboratory experiment and field test, future research should cover the whole chain of process development in an integrated and iterative way [6]. In this context, selection and assessment of robust microalgal strains for large-scale cultivation have become one of the key research topics for biodiesel production. Small-scale open systems should be used as selection devices for microalgae strains suitable for outdoor mass culture [13]. Full-scale assessment of lipid-rich microalgae should be performed not only under the laboratory conditions, but also in the fields, to test their adaptability to changes in temperature, strong light irradiance, and other chemical and biological environment conditions.

*Graesiella* sp. WBG-1 is a unicellular green microalga with broadly ellipsoidal or globose cells. This strain was originally isolated from Chenghai Lake, Yunnan province, China, by the researchers in our lab. Molecular analysis contributed the WBG-1 strain mainly to genus *Graesiella* and showed 99.8 % similarity with two *Graesiella* species: *Graesiellaemersonii* and *Graesiellavacuolata* based on the 18S rDNA/ITS sequence. Preliminary investigations using a bubbled column photobioreactor showed *Graesiella* sp. WBG-1 to be one of the most productive microalgae among the 63 studied Chlorophyta strains (Additional file 1). Furthermore, this strain also possesses some other desirable features, such as large cell size (easy to harvest) and high adaptive capacity to a wide range of culture pH. All the above advantages together encouraged us to perform a comprehensive selection and assessment using this promising strain *Graesiella* sp. WBG-1.

Given the above considerations, we carried out both indoor and outdoor experiments to study the effects of several fundamental factors on the growth and lipid accumulation of the microalga *Graesiella* sp. WBG-1, and to test its robustness under the outdoor environmental conditions in a 200 m^2^ raceway pond. CO_2_ utilization and other features of *Graesiella* sp. WBG-1 were also discussed.

## Methods

### Growth medium and preparation

The basal growth medium for microalgae culture was a modified BG-11 medium which had the following components (per liter): NaNO_3_ (100 mg), K_2_HPO_4_·3H_2_O (40 mg), MgSO_4_·7H_2_O (75 mg), CaCl_2_·2H_2_O (36 mg), citric acid (6 mg), Fe-ammonium citrate (6 mg), EDTA·Na_2_ (1 mg), NaHCO_3_ (20 mg), H_3_BO_3_ (2.86 mg), MnCl_2_·4H_2_O (1.8 mg), ZnSO_4_·7H_2_O (0.22 mg), CuSO_4_·5H_2_O(0.08 mg), Na_2_MoO_4_·2H_2_O (0.391 mg), and Co(NO_3_)_2_·6H_2_O (0.0494 mg). Sodium nitrate and sodium bicarbonate concentrations were modified as indicated in the text.

The BG-11 medium was prepared with deionized water for laboratory experiments, and with water from Chenghai Lake (containing ~1 g L^−1^ NaHCO_3_) for outdoor experiments. The deionized water was sterilized by autoclave, and the water from Chenghai Lake was purified by filtration, and then, the sterile nutrient stock solutions were added to either the deionized water or the water from Chenghai Lake to make final medium. The culture pond was exposed to direct solar irradiation for at least 12 h before medium preparation.

### Bubbled column PBR cultivation

Each column of the bubbled column PBR used in this study has an inner diameter of 3 cm and a working volume of 200 mL. The columns were illuminated with white fluorescent tubes for 14 h every day, and the light intensity at the reactor surface was 300 μmol m^−2^ s^−1^ (15 mol photons m^−2^ day^−1^). CO_2_-enriched (1 %, v/v) air was injected at the bottom of the column through a glass tube, that is used for CO_2_ supplement, culture mixing, and O_2_ exchange. The air flow rate was maintained at 250 mL min^−1^. A thermostatic water circulator was used as water bath to keep the temperature of the culture columns at 30 °C.

The algal cells were harvested from seed culture and resuspended into sterilized BG-11 medium for inoculation. The cells were grown in batch with an initial cell density of 0.5 ± 0.05 (optical density at 540 nm). To investigate the effects of nitrate and bicarbonate concentration on cell growth and lipid accumulation, different doses of sodium nitrate or sodium bicarbonate were added into each column before inoculation. All of the experiments were carried out in triplicate.

### Scale-up cultivation in a 10 L circular pond

A mini circular pond cultivation system was used to simulate the outdoor open pond cultivation under artificial illumination. The circular pond was equipped with four units to control mixing, irradiance, temperature, and pH, as described in previous study [14].

The cells were grown in batch with an initial cell density of 0.1 ± 0.05 (optical density at 540 nm). Total culture volume was about 10 L giving a culture depth (light path) of 10 cm, and the effective illuminated area was 0.1 m^2^. Banks of white fluorescent tubes were placed above the pond, and provided 300 µmol photons m^−2^ s^−1^ with 14 h:10 h light–dark cycle (15 mol photons m^−2^ day^−1^). The cultures were kept at 30 °C and mixed at 50 r min^−1^ continuously. Pure CO_2_ was dispersed into the culture suspension through a gas diffuser under the programming control of an online pH sensor, to maintain the culture pH within a desired range. In another study, we found that *Graesiella* sp. WBG-1 was well adapted to culture pH range of 7–10 (unpublished data). Therefore, the culture pH of 9.0 ± 0.5 was maintained in the simulate experiments to facilitate external CO_2_ uptake. The experiments were carried out in duplicate.

### Scale-up cultivation in a 30 L tank PBR

To test the adaptability of the strain to variable environmental conditions, outdoor scale-up cultivations of the *Graesiella* sp. WBG-1 were carried out in 30 L tank PBR under natural light illumination.

The PBR used in this experiment consisted of four open-top polyethylene tanks placed side-by-side in a single row on a concrete platform. The working volume is 30 L for each culture tank, giving an illuminated area of 0.19 m^2^. An electromagnetic air compressor continuously blew sterilized air into the culture suspension during cultivation. The air flow was maintained at 3 L min^−1^. The air was enriched with CO_2_ (1 %, v/v) during light period to support cell growth and maintain pH within the desired range (9.0 ± 0.5). Natural solar irradiance and environmental temperature were monitored and logged on site with an automatic weather station.

### Outdoor cultivation in 200 m^2^ raceway pond

Pilot-scale evaluation of *Graesiella* sp. WBG-1 was carried out at Chenghai Lake, Yunnan province, China, (N26°29′29.64″ E100°40′56.12″) using a traditional raceway pond.

The raceway pond was an open system constructed of concrete blocks. The pond was 20 m long, 12 m wide, giving an effective culture area of 200 m^2^. Two large paddle wheels were installed apart within the raceway for mixing, and were driven by speed-adjustable motors and able to provide a flow velocity of 10–60 cm s^−1^. The liquid flow velocity was set at 45 cm s^−1^ in this study, and the paddle wheels were turned on in daytime and turned off in night. Pure CO_2_ was automatically injected into the culture via a 3 m-long microporous polymer tube (gas diffuser) that was placed at the bottom of the culture pond. The automatic injection of CO_2_, which was controlled by an online pH sensor, maintained the culture pH within the desired range (pH 9.0 ± 0.5). The working culture depth (light path) was 20 cm, corresponding to 40,000 L culture volume. Natural solar irradiance, environmental temperature, and suspension temperature were monitored and logged on site with an automatic weather station.

Two similar but smaller raceway ponds (20 m^2^) covered by greenhouse were used for high-quality seed culture preparation. Independent batch cultures in the 200 m^2^ pond were carried out three times in June 2013, July 2013, and May 2014.

### Analytical procedure

Culture growth was estimated by measuring the dry biomass concentration of the culture broth. About 10 mL culture broth was filtered through a pre-dried GF/C glass microfiber filter paper (pore size 0.45 µm), and dried at 105 °C for 4 h, and then weighed to calculate dry biomass concentration (DW, in grams of biomass per liter of culture broth). Daily biomass productivity was calculated by dividing the difference between the DWs at the start time and the end time by its duration (days). The microalga was stained with Nile Red according to Li’s method [10] and observed using a Nikon Eclipse 80i microscope.

A spectrophotometric method, described by Collos et al. [15], was used to monitor residual nitrate concentration. Briefly, the absorbance of culture filtrate (0.22 µm filter) at 220 nm and a pre-constructed standard curve were used to determine residual nitrate.

Cells were collected by centrifugation (5000 rpm for 5 min) and lyophilized (−56 °C cryotrapping, 10–14 Pa vacuum) for biochemical analysis. For lipid quantification, 50 mg of dry algal biomass was fully grounded, transferred to a covered centrifuge tube, and then extracted with a mixture of *n*-hexane and ethyl acetate (1:1, v:v) for 20 min. The extraction was repeated three times, and all extracts were combined into a pre-weighed glass tube, and then dried under nitrogen protection. The lipids were determined gravimetrically.

Neutral lipid (Triacylglycerides, TAGs) was fractionated from the lipid extracts by column chromatography using a 2 cm × 20 cm column packed with 4 g silica gel 60 [16]. The lipid extracts (~100 mg) were dissolved in 2 mL chloroform and loaded onto the column. TAGs were eluted from the column by 20 mL chloroform. The eluted TAGs fraction were confirmed by TLC, and then dried and quantified gravimetrically.

To analyze fatty acids, about 20 mg of the lipid extract was dissolved in 3 mL *n*-hexane and then transmethylated by adding 3 mL methanol-KOH (0.5 % KOH) and heating at 50 °C for 60 min. After cooling to room temperature, the hexane layer was separated and dried with anhydrous sodium sulfate. Fatty acid methyl esters were analyzed by gas chromatography (Agilent 7890A) using an HP-5 Phenyl Methyl Siloxan column (30 m × 0.32 mm × 0.25 µm) and a flame ionization detector. 1 µL fatty acid methyl esters solution was injected to the sampler with a splitting ratio of 5:1. The heating program was 150 °C held for 2 min, then increased to 250 °C at a rate of 10 °C per min, and held for 8 min. A standard FAME Mix (Sigma-Aldrich) was used for fatty acid identification.

About 10 mg of lyophilized algal powder was used for total carbohydrate quantification. The algal powder was transferred to a covered tube, fully mixed with 1 mL hydrochloric acid (6 M), to digest at 105 °C for 1 h. After cooling to RT, about 1 mL NaOH (6 M) was added into the solution to neutralize acid, followed by centrifugation at 3500*g* for 5 min. The supernatant was collected into a new tube and its volume was brought to 2 mL with deionized H_2_O. 100 µL of the diluted supernatant was diluted again with deionized H_2_O to 2 mL, mixed with 1 mL phenol (6 %), and then 5 mL sulfuric acid was trickled into the sample for color development. Finally, the optical density at 490 nm was measured on a spectrophotometer. To quantify total carbohydrate content, glucose was used to establish the standard curve.

Protein content was determined as described by Slocombe et al. [17]. Briefly, 10 mg freeze-dried algal powder was suspended in 500 µL of 24 % (w/v) trichloroacetic acid (TCA) and then incubated at 95 °C for 15 min. The lysate containing 24 % (w/v) TCA were cooled to RT and diluted to 6 % (w/v) with 1.5 mL deionized water. The homogenate was centrifuged at 15,000*g* for 20 min and the supernatant was discarded. The pellets were resuspended in 1 mL NaOH (1 M) by repeated pipetting and then incubated at 40 °C for 2 h. The protein concentration was then spectrophotometrically measured according to standard Bradford assay.

### Calculations

Areal biomass concentration (*C*_biomass_, g m^−2^) was calculated by the following:

1 $$C_{\text{biomass}} = \frac{{{\text{DW}} \times V}}{S}$$

with DW as measured biomass dry weight (g L^−1^), *V* as culture volume (L), and *S* as illuminated area (m^2^).

Biomass productivity (*P*_biomass_, g m^−2^ day^−1^) was calculated according to the following:

2 $$P_{\text{biomass}} = \frac{{C_{{{\text{biomass}},t2}} - C_{{{\text{biomass}},t 1}} }}{t2 - t1 \, }$$

with *C*_biomass,*t*2_ and *C*_biomass,*t*1_ as biomass concentrations at culture time *t*2 (day) and *t*1 (day), respectively.

Lipid productivity (*P*_lipid_, g m^−2^ day^−1^) was calculated according to the following:

3 $$P_{\text{lipid}} = \frac{{C_{{{\text{biomass}},t2}} \times C_{{{\text{lipid}},t2}} - C_{{{\text{biomass}},t 1}} \times C_{{{\text{lipid}},t1}} }}{t2 - t1 \, }$$

with *C*_lipid,*t*2_ and *C*_lipid,*t*1_ as lipid content at culture time *t*2 (day) and *t*1 (day), respectively.

Average daily light intensity (*I*_av_, mol m^−2^ day^−1^) was calculated according to the following:

4 $$I_{\text{av}} = \frac{{\sum\nolimits_{0}^{t} {I_{\text{inc}} } }}{t \, }$$

with *I*_inc_ as daily incident light intensity (mol m^−2^ day^−1^) measured by the automatic weather station.

Biomass-specific light availability (*I*_biomass_, mol g^−1^ day^−1^) was calculated according to Ref. [18] by the following:

5 $$I_{\text{biomass}} = \frac{{I_{\text{av}} }}{{C_{\text{biomass}} \, }}$$

CO_2_ utilization rate (*R*_c_, %) was calculated by the following:

6 $$R_{\text{c}} = \frac{{W_{\text{b}} \times 1000 \times 0.5 \div 12}}{{C_{1} - C_{2} + C_{3} }} \times 100\,\%$$

with *W*_b_ as net increase of biomass (kg) during cultivation; *C*_1_ as dissolved inorganic carbon ([H_2_CO_3_] + [CO_3_^2−^] + [HCO_3_^−^], mol) in the medium at the begin of culture; *C*_2_ as dissolved inorganic carbon (mol) in the medium at the end of culture; and *C*_3_ as the total CO_2_ (mol) used during culture. The bio-fixated carbon (50 % of the biomass is *C*) divided by consumed carbon was defined as CO_2_ utilization rate in this study.

## Results

### Effects of nitrate and bicarbonate on cell growth of *Graesiella* sp. WBG-1 under the laboratory conditions

The microalga *Graesiella* sp. WBG-1 was cultivated in a 200 mL column photobioreactor under different bicarbonate and nitrate concentrations, to study the effects of carbon and nitrogen on the cell growth and lipid accumulation, which were essential for mass production optimization.

**Fig. 1:**
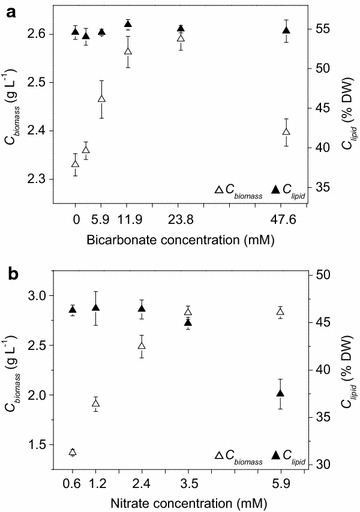
Effects of bicarbonate (**a**) and nitrate (**b**) on cell growth (*open triangle*) and lipid accumulation (*filled triangle*)

As shown in Fig. 1a, the biomass concentration (*C*_biomass_) of *Graesiella* sp. WBG-1 increased almost linearly with increasing concentrations of bicarbonate from 0 to 11.9 mM, and the increase was no more significant when the concentration of bicarbonate was above 11.9 mM. When the concentration of bicarbonate was increased to 47.62 mM, the *C*_biomass_ of *Graesiella* sp. WBG-1 dropped significantly due to excessive salinity. In contrast, the lipid content (*C*_lipid_) of *Graesiella* sp. WBG-1 did not change under all tested bicarbonate concentrations (Fig. 1a).

The effects of nitrate on cell growth and lipid accumulation were shown in Fig. 1b. The *C*_biomass_ of *Graesiella* sp. WBG-1 increased with increasing nitrate concentrations, while the lipid content (*C*_lipid_) decreased gradually (Fig. 1b). The maximum *C*_biomass_ of ~2.8 g L^−1^ was achieved at 5.88 mM nitrate, while the *C*_lipid_ further decreased to 37.46 % DW at this concentration.

### Lipid production of *Graesiella* sp. WBG-1 in small-scale reactor

**Fig. 2:**
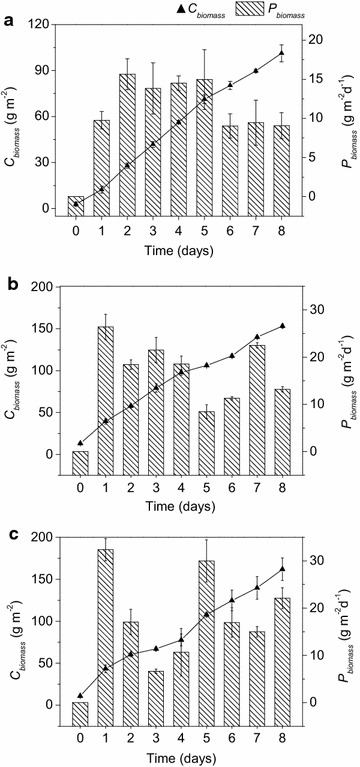
Microalgal growth and biomass productivity in small-scale reactor. **a** Cultivation in 10 L laboratory circular pond; **b** cultivation in 30 L outdoor tank PBR in June 2012; and **c** cultivation in 30 L outdoor tank PBR in July 2012. Time evolution of biomass concentrations and biomass productivities are indicated by *line* and *column,* respectively. The areal biomass concentration (*C*_biomass_, g m^−2^) was used in all outdoor experiments

As shown in Fig. 2a, in a 10 L circular pond, *Graesiella* sp. WBG-1 showed fast and continued growth (*C*_biomass_) during 8 days of cultivation under 15 mol m^−2^ day^−1^ artificial light irradiation. Although the nitrate in the algal suspension got depleted from day 2 (Additional file 2), the cells still kept fast growth (*C*_biomass_) and the growth slightly slowed down from day 5. The overall biomass productivity (*P*_biomass_) was 12.03 g m^−2^ day^−1^. From day 2 to day 5, the biomass productivities maintained at ~14 g m^−2^ day^−1^. The maximum biomass productivity (15.65 g m^−2^ day^−1^) was achieved on day 2.

Cultivation of *Graesiella* sp. WBG-1 in 30 L tank PBR was carried out twice under direct sun light irradiation. The first cultivation (Fig. 2b) was carried out in June 2012 with an average light intensity of 28 mol photon m^−2^ day^−1^ and air temperature of 23.2 °C. The second time (Fig. 2c) was carried out in July 2012 with an average light intensity of 31 mol photon m^−2^ day^−1^ and air temperature of 24.8 °C.

For both cultivations, no obvious adaptation phase was observed in the 30 L tank PBR, and the *C*_biomass_ increased continuously with slight fluctuation in growth rate, perhaps, due to fluctuating natural irradiance (Additional file 3). The biomass productivity fluctuated even more through time with fluctuating light. At the end of cultivation, very similar *C*_biomass_ (~155 g m^−2^) was achieved in both experiments. The average *P*_biomass_ was 17.53 g m^−2^ day^−1^ for the first cultivation (June 2012) and 18.85 g m^−2^ day^−1^ for the second cultivation (July 2012), respectively.

The seed *Graesiella* sp. WBG-1 cultured under nitrate sufficient condition only contained a small proportion of lipids (18.01 % DW), of which 47.22 % were TAGs. After 8 days of cultivation in the 10 L circular pond, the *C*_lipid_ increased to 45.23 % DW and over 86 % of the lipids were TAGs (Table 1). Compared to the small-scale cultivation, lower *C*_lipid_ was achieved in 30 L outdoor cultivations, partly because of lower availability of biomass-specific light (Discuss in detail later). However, the lipid productivity (*P*_lipid_) achieved in outdoor 30 L cultivations was higher than those of 10 L cultivations, because more biomass (though containing lower lipid) was produced per illuminated area.

**Table 1:** Lipid production in the two small-scale reactors and corresponding light conditions

Trials	*I*_biomass_ (mol g^−1^ DW day^−1^)	*C*_lipid_ (% DW)	TAGs (% DW)	*P*_lipid_ (g m^−2^ day^−1^)
10 L	0.2–5.3	45.2 ± 1.2	39.3 ± 0.5	5.7 ± 0.8
30 L (June 2012)	0.2–2.1	35.6 ± 1.0	30.8 ± 1.0	6.5 ± 0.8
30 L (July 2012)	0.2–2.8	32.9 ± 1.3	30.0 ± 0.4	6.4 ± 1.2

### Cultivation of *Graesiella* sp. WBG-1 in outdoor 200 m^2^ raceway pond

**Fig. 3:**
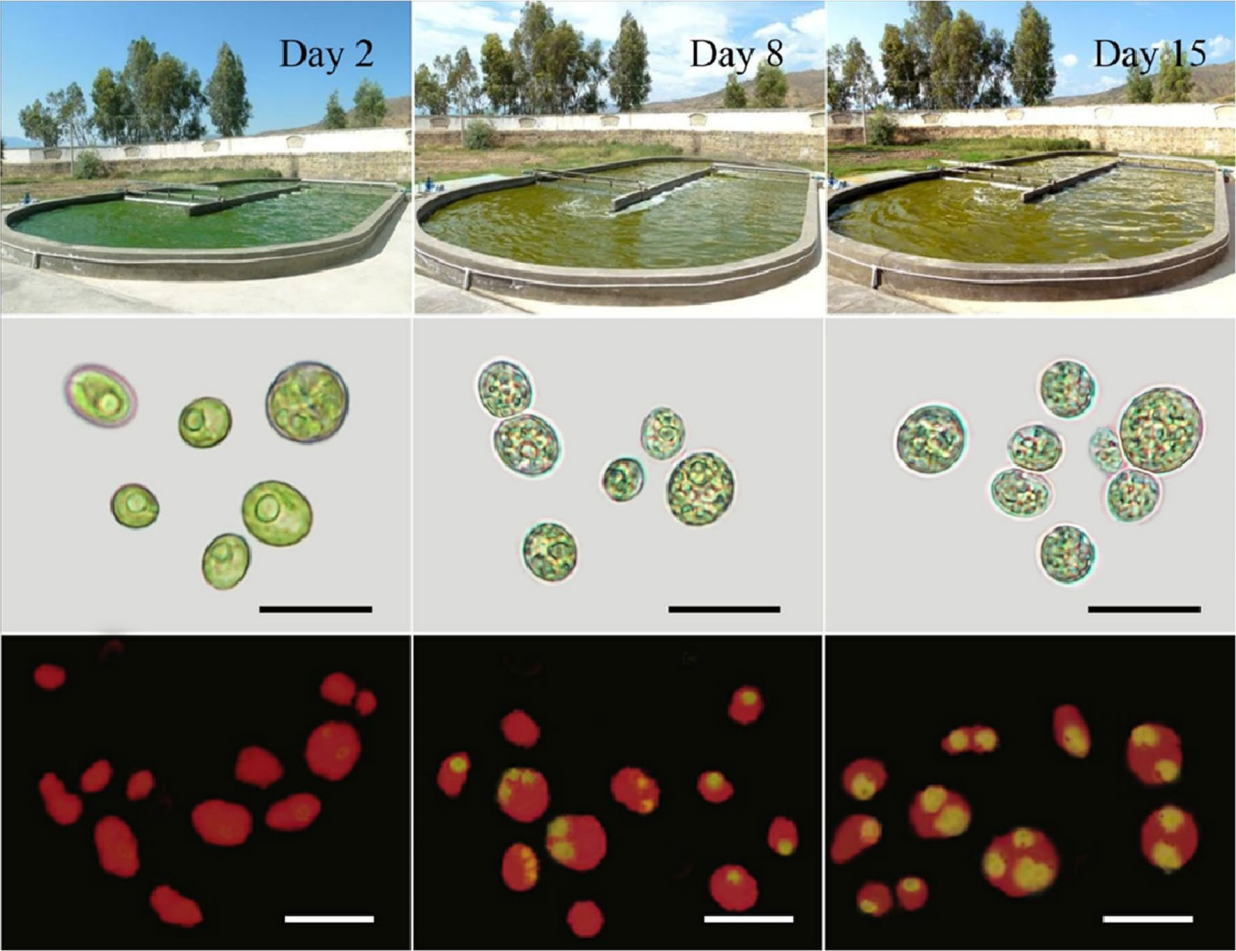
Cultivation of *Graesiella* sp. WBG-1 in 200 m^2^ raceway pond. Changes in culture color and morphology of the cells during cultivation were shown. Intracellular lipid bodies were stained using Nile Red (*yellow*) and auto-fluorescence of chloroplasts is seen in *red*. *Scale bar* 10 µm

Three batch cultures of the *Graesiella* sp. WBG-1 in a 200 m^2^ raceway pond were carried out three times in June 2013, July 2013 and May 2014. Figure 3 shows the changes of the culture color as well as the morphologic appearance of the cells. At the early stage of the batch culture, the microalgae suspension looked dark green and a pyrenoid could be observed in the cell. With the aging of culture, the pyrenoid was disappeared and the culture color was yellowish. After 15 days of cultivation, yellow-green or orange color could be seen in the raceway pond.

**Fig. 4:**
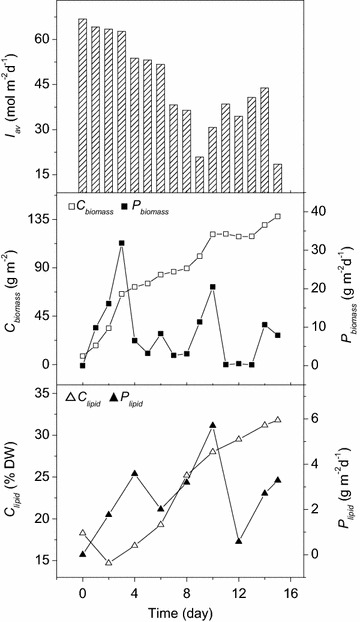
Time evolution of biomass concentration (*C*_biomass_, *open square*), biomass productivity (*P*_biomass_, *filled square*), lipid content (*C*_lipid_, *open triangle*), lipid productivity (*P*_lipid_, *filled triangle*), and natural light intensity (*I*_av_) during the outdoor raceway cultivation (June 2013)

The time evolution of biomass concentration, lipid productivity, as well as natural light intensity, during the first cultivation in June 2013, were shown in Fig. 4 as a typical example, and a summary (*C*_biomass_, *C*_lipid_, *P*_biomass_, *P*_lipid_, et al.) of the three cultivations was illustrated in Table 2.

**Table 2:** Summary of the cultivations at different scale

Cultures	Duration (days)	*I*_av_ (mol m^−2^ day^−1^)	*I*_biomass_ (mol g^−1^ DW day^−1^)	*C*_biomass_ (g m^−2^)	*C*_lipid_ (% DW)	*P*_biomass_ (g m^−2^ day^−1^)	*P*_lipid_ (g m^−2^ day^−1^)
10 L circular pond	8	15	0.2–5.38	101.2	45.2	12.0	5.7
30 L tank (June 2012)	8	33	0.2–2.1	153.3	35.6	17.5	6.5
30 L tank (July 2012)	8	29	0.2–2.8	161.8	32.9	18.9	6.4
Raceway (June 2013)	15	45	03–5.7	137.8	31.8	8.7	2.9
Raceway (July 2013)	13	36	0.3–4.4	102.3	33.4	7.2	2.4
Raceway (May 2014)	14	39	0.4–3.4	97.7	29.4	6.2	2.0

As shown in the middle panel of Fig. 4, the microalga grew rapidly in the first 4 days of cultivation in the raceway pond, and the *C*_biomass_ increased from 7.86 to 65.67 g m^−2^. After day 4, the cell growth slowed down, because the nitrate in the algal suspension was depleted (Additional file 2) and the natural light intensity was reduced (upper panel of Fig. 4).

As shown in the lower panel of Fig. 4, the *C*_lipid_ of *Graesiella* sp. WBG-1 decreased in the first 2 days of cultivation and then increased afterwards, from 14.70 to 31.82 % DW. The *P*_lipid_ showed positive correlation with the *P*_biomass_, suggesting the essential role of biomass productivity in lipid production. Fluorescence microscopic observation (Fig. 3) also demonstrated the increase in lipid content in *Graesiella* sp. WBG-1 cells.

**Fig. 5:**
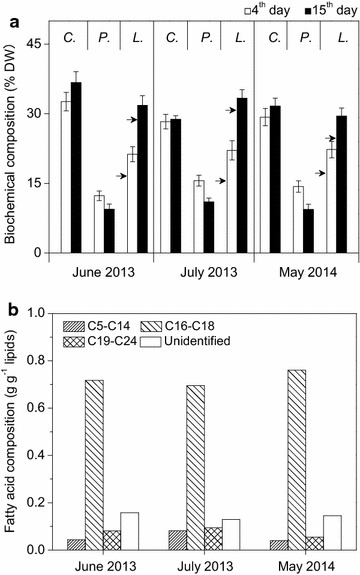
Biochemical compositions (**a**) and fatty acid profiles (**b**) of *Graesiella* sp. WBG-1 in 200 m^2^ raceway cultivation. Carbohydrate, protein, and lipid at the 4th day (*open column*) and 15th day (*filled column*) are denoted with *C.*, *P.,* and *L.,* respectively. *Arrows* indicate TAGs content on dry biomass bases (% DW)

The biochemical compositions and fatty acid profiles of *Graesiella* sp. WBG-1 in 200 m^2^ raceway cultivation were also examined on the day 4 and day 15 of cultivation, when the cells were under the nitrate starvation condition. The carbohydrate content as a percentage of dry biomass did not show a significant change in all three experiments, while the protein content decreased with aging of the culture (Fig. 5a). A significant increase was observed in total lipids, especially the storage TAGs (Fig. 5a; Table 2), which made up 28.76 % of the dry biomass on the day 15. In contrast, the storage TAGs on the day 4 were only made up 16.67 % of the dry biomass. The fatty acid profile was analyzed using total lipids instead of TAGs, because DAGs and other free fatty acids in the total lipids can also be used to form fatty acid methyl esters. As shown in Fig. 5b, the most abundant fatty acids in *Graesiella* sp. WBG-1 cultivated in the 200 m^2^ raceway pond were C16–C18 (especially C16:0 and C18:1), and the amounts of C5–C15 and C1–9C24 fatty acids were relatively low.

## Discussion

### Optimization of nitrate and bicarbonate concentration

Nitrogen deficiency is the primary stress to alter algal metabolism to TAG synthesis, while limit cell growth at the same time [8, 19]. These general trends were also observed in *Graesiella* sp. WBG-1, as shown in Fig. 1b. Adams et al. [20] pointed out that culturing either many cells with low lipid content or few cells with high lipid content will not result in an economically viable biodiesel feedstock, although both cases give high lipid productivity. In this sense, the initial nitrate concentrations of 0.59 and 5.88 mM, in this study (Fig. 1b), will not suitable for microalgal lipid production. The optimal nitrate concentration range for lipid production should be 1.18–3.53 mM in this study (Fig. 1b) to balance the trade-off between the growth and lipid accumulation of *Graesiella* sp. WBG-1.

**Fig. 6:**
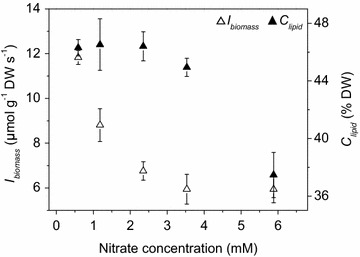
Lipid content (*C*_lipid_, *filled triangle*) and biomass-specific light availability (*I*_biomass_, *open triangle*) as functions of nitrate concentration

On the other side, initial nitrate concentration of a simple bath culture also put strong influence on biomass density. To some extent, the higher the initial nitrate concentration, the higher the biomass density could be achieved (Fig. 1b). However, when biomass density is low, light has good penetration and individual cells are exposed to a large quantity of light energy [18], resulting in more metabolic carbon flux to be channeled to lipid accumulation [21, 22]. Our observation was consistent with these conclusions, showing that low nitrate concentration not only resulted in high *C*_lipid_, but also low biomass density (Fig. 1b), which helped to form high light availability (*I*_biomass_) in the culture (Fig. 6). *I*_biomass_ was in turn favorable for high lipid content (*C*_lipid_) [18]. Therefore, the optimal nitrate concentration should be able to give sufficient light availability per gram of biomass.

*I*_biomass_ varies depending on reactor setup as well as light intensity and initial nutrition. A rough simulation revealed a huge difference in *I*_biomass_ among four reactors used in this study (Additional file 4). Given the same volumetric biomass concentration, *I*_biomass_ in 200 mL column was four times higher than that in the outdoor 30 L tank PBR, and *I*_biomass_ in 30 L tank PBR and 200 m^2^ raceway pond were very similar. Thus, compared to the cultivation in 200 mL column, a lower biomass concentration (*C*_biomass_) is needed for 30 L tank PBR and 200 m^2^ raceway pond, to give a relatively higher *I*_biomass_ to facilitate lipid accumulation. Therefore, among the optimal nitrate concentration range of 1.18–3.53 mM for lipid production, 1.18 mM nitrate is the best concentration for following outdoor cultivation. By sacrificing some of the algal growth using reduced initial nitrate concentration, fairly high light availability (*I*_biomass_) could be achieved.

Several studies have demonstrated the importance of photosynthesis efficiency on oil formation [18, 21, 23]. In other words, normal photosynthesis is needed to produce enough carbon flux and drive the biosynthesis of TAGs under nitrogen stress. Actually, carbon in cultures with air-blowing or paddle wheel stirring is far from sufficient [24, 25]. Thus, addition of carbon is another possible way to improve microalgal lipid production. White et al. [26] reported that addition of bicarbonate could significantly increase cell abundance in culture of *Tetraselmis suecica* and *Nannochloropsis salina*. We got a similar result using *Graesiella* sp. WBG-1 (Fig. 1a), although the *C*_biomass_ were statistically insignificant between 11.90 and 23.81 mM bicarbonate. Lipid accumulation, however, was unaffected by different bicarbonate concentrations in this study. The optimal bicarbonate concentration for *Graesiella* sp. WBG-1 cultivation, therefore, is 11.90 mM considering the culture cost.

### Performance of *Graesiella* sp. WBG-1 in scale-up cultivation

The 10 L photobioreactor used in this study is a mini-type industrial circular pond, equipped with light, temperature, and pH control systems. In this reactor, the strain *Graesiella* sp. WBG-1 showed good growth (12.03 g m^−2^ day^−1^*P*_biomass_) and high *C*_lipid_ (45.23 % DW) (Table 2), consistent with the previous results observed in our laboratory. Compared to the 10 L photobioreactor, higher *P*_biomass_ (17.53–18.85 g m^−2^ day^−1^) was achieved in outdoor cultivation in 30 L tank PBR. However, the *C*_lipid_ (32.89–35.63 % DW) decreased in 30 L tank PBR. The decrease in lipid content could be partly interpreted as a result of lower availability of biomass-specific light (*I*_biomass_, the average light irradiance received by each gram of biomass) [18]. Light is an important stress factor for microalgal lipid accumulation [8]. It is also reported that the de novo synthesis of intracellular TAGs is highly correlated with photosynthetic activity as well as light energy available for microalgal cells [21, 23]. In this sense, sufficient light supply (higher *I*_biomass_) is helpful to get a high lipid content.

In fact, the 30 L tank PBR has a longer light path (15 cm) than the 10 L circular pond (10 cm), giving a much lower biomass-specific light availability (*I*_biomass_). Although lower *C*_lipid_ was achieved in 30 L tank PBR, *P*_biomass_ was significantly higher than achieved in 10 L circular pond, because there were more growing cells per illuminated area in the 30 L tank PBR. As a result, higher lipid productivity was achieved in the 30 L tank PBR. On the other hand, outdoor light irradiance was fluctuated in a wide range of 16–44 mol m^−2^ day^−1^ during the experiments (Additional file 3), and the highest instant light irradiance reached 2500 μmol m^−2^ s^−1^, but no lag phase was observed in both laboratory and outdoor conditions. These results indicated the nice adaptability of *Graesiella* sp. WBG-1 to strong and fluctuating natural solar irradiance.

Cultivation of *Graesiella* sp. WBG-1 in 200 m^2^ raceway pond was successfully carried out three times in the summer at Chenghai town, where most days are sunny throughout the spring and early summer. The average daily light intensity during these three cultivations was 45, 36, and 39 mol m^−2^ day^−1^, respectively, which was two-to-three times as high as the artificial light intensity in the 10 L circular pond. Under such light irradiance, the daily average *P*_biomass_ achieved in 200 m^2^ raceway pond was 6.17–8.66 g m^−2^ day^−1^, substantially lower than the productivity previously reported in studies of microalgal mass production in food/feed industry [27]. Nevertheless, a maximum *P*_biomass_ of 31.86 g m^−2^ day^−1^ in 200 m^2^ raceway pond (Fig. 4) was reached in the first 3 days when nitrate was sufficient. The marked difference between average *P*_biomass_ and maximum *P*_biomass_ suggested that the cell growth was severely limited by nitrate depletion in the later 12 days of cultivation. Actually, the initial nitrate concentration was only 1.18 mM, which was insufficient for the alga to grow 15 days in 200 m^2^ raceway pond. On the other hand, lower initial nitrate concentration only supported lower *C*_biomass_, and thus resulted in higher *I*_biomass_, which was found to promote lipid accumulation [18]. As a result, relatively high *C*_lipid_ (29.41–33.42 % DW) was achieved in the 200 m^2^ raceway pond. If the initial nitrate concentration was increased from 1.18 to 1.76 mM, no increase in the *C*_lipid_ of *Graesiella* sp. WBG-1 could be observed after 15 days of cultivation in raceway pond (unpublished data). Therefore, the biological processes of cell growth and lipid accumulation should be further studied to optimize momentum, energy, and mass transfer in raceway pond.

Although the lipid content of 29.41–33.42 % DW is much lower than achieved in the small-scale cultivation (nitrate-deplete) in laboratory, it is of great importance for two reasons. First, Chlorophyta strains are reported to have a *C*_lipid_ of 10–25 % DW under nitrate-replete conditions [28]. For the microalga *Graesiella* sp. WBG-1, the *C*_lipid_ is about 14–17 % DW under nitrate-replete conditions (Fig. 4). The final *C*_lipid_ of 29.41–33.42 % DW obtained in the 200 m^2^ raceway pond under nitrate-depleted conditions is nearly two times the initial value. Furthermore, TAGs contributed a large proportion (84.38–91.88 %) of the total lipids in the harvested cell mass, and C16–C18 were the major fatty acids, comprising more than 70 % of total fatty acids. These data clearly indicated that lipid was markedly accumulated in the cells of *Graesiella* sp. WBG-1 cultured in the 200 m^2^ raceway pond.

Second, sharp declines in *C*_lipid_ with increasing culture volumes have been observed in many studies. For example, Li et al. [10] reported that *Parachlorella kessleri* CCALA 255 had a lipid content of 55 % DW in laboratory, but only 25 % DW when cultured in a Large-Scale Industrial Thin-Layer Bioreactor. In a 2000 L raceway pond, the green microalga *Botryococcus bruanii* AP103 reached a total lipid content of only 30.8 % DW, after 15 days of outdoor cultivation [11]. More recently, more than 50 % decrease in lipid content (from 40 to ~19.8 % DW) was observed in *Chlorella* strain CH2, when its cultivation was scaled up from 0.25 GWP to 1 ha GWP [12]. In other studies in our lab, although some strains of *Chlorella* and *Scenedesmus* can accumulate large amounts of lipids (over 50 % DW) in laboratory, their lipid content are similar to the initial values (~20 % DW), and there is almost no net increase in TAGs when cultivated in the raceway pond (Additional file 5) with the same technology described in this study. However, the decline in *C*_lipid_ of the microalga *Graesiella* sp. WBG-1 cultured in the 200 m^2^ raceway pond is less than the other tested microalgae. Moreover, more than one-fold increase in TAGs was observed. These phenomena suggest that lipid accumulation does not always occur for any oleaginous microalgae in mass open culture. One possible explanation is the loss of culture competitiveness [13]. This is why the field test is necessary for oleaginous microalgae selection. As discussed above, *Graesiella* sp. WBG-1 is a potential oil producer with wide tolerance to natural environmental conditions.

### Other features of *Graesiella* sp. WBG-1 for large-scale cultivation in open ponds

**Fig. 7:**
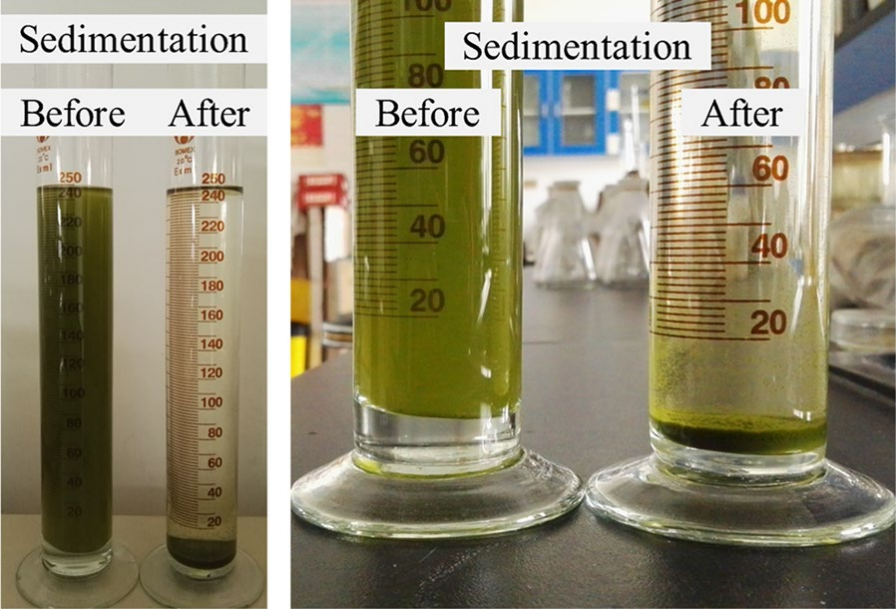
Sedimentation of *Graesiella* sp. WBG-1 cells over night. The homogeneous cell suspension (*left*) was kept still in a glass cylinder and over 98 % of biomass sedimented to the bottom after about 8 h (*right*)

As indicated in many studies, lipid productivity is not the sole criterion for the selection of lipid-rich microalgae. One of the desirable characteristics for mass culture is large cell size [28], which is assumed to be easier to sediment in liquid culture [29] and reduce harvesting and down-stream processing costs. *Graesiella* sp. WBG-1 cells are broadly ellipsoidal or globose with a diameter of 8–12 µm, which is significantly larger than other reported oleaginous microalgae, such as *Chlorella* (diameter 3–8 µm). It was observed in this study that *Graesiella* sp. WBG-1 cells settle down easily in medium in a short time, and this feature becomes more obvious with the aging of culture. The high culture pH (9.0 ± 0.5 in this study) may also play a role in the auto-participation [30]. Over 98 % of the cells settled down to the bottom of the raceway pond when the algal suspension was kept without stirring over night. By removing the upper layer medium from the pond, the algal biomass was concentrated more than 50 times (Fig. 7). This feature potentially reduce liquid volume for harvesting if a standing procedure is employed before centrifugation or filtration, and the harvesting cost is reduced then.

Biological contamination is a serious problem for microalgae cultivation in open pond [31]. Protozoa may be predators on algae, bacteria and fungi, may be parasites, and other non-target microalgae may compete with the cultured algae for light and nutrients [32]. Theoretically, the ability to resist contamination is different among different algal strains, because there are many factors that can potentially inhibit the growth of contaminated organism, such as microalgal cell size and their tolerance to extreme environmental conditions, including temperature, pH, and salinity. Recently polysaccharides from algae are reported to have antibacterial activity [33], suggesting allelopathy is a prospective way for contamination control in microalgae mass cultivation [34]. In our experience, *Graesiella* sp. WBG-1 is more resistant to contamination compared to other tested algae. Although invading organisms, such as bacteria and zooplankton, could be identified under microscope in *Graesiella* sp. WBG-1 culture, but the number of these organisms was limited and the negative effects on productivity was negligible. In contrast, zooplankton contamination often led to culture collapse in the open cultivation of *Chlorella*, *Scenedesmus*, *Desmodesmus,* and *Chlorococcum*. It is not clear if *Graesiella* sp. WBG-1 has some specific chemical defense system or not, but *Graesiella* sp. WBG-1 is an indigenous strain isolated from Chenghai Lake and well adapted to the environment. Maybe, its large cell size can cause swallowing difficulty for protozoa.

Coupling bio-fixation of carbon dioxide with microalgae triacylglycerides production is a hot research topic worldwide [35, 36]. It is known that sufficient gas–liquid contact is important for efficient absorption of CO_2_ [36], and high pH can prevent the escape of CO_2_ from culture [37]. In this study, a microporous tubular gas diffuser (average pore size <100 µm) was used to maximize CO_2_-water contact, and a high culture pH (9.0 ± 0.5), which had no negative effects on *Graesiella* sp. WBG-1 growth and lipid accumulation (unpublished data), was maintained in 200 m^2^ raceway to prevent the escape of CO_2_. Under such condition, a CO_2_ utilization rate (bio-carbon divide by consumed carbon) of 65.7 % was achieved. This utilization rate is even higher than that those achieved in the mass culture of *Spirulina* (60 %) [38, 39], *Chlorella* (46 %) [40], and *Phaeodactylum* (63 %) [41]. These results indicate that *Graesiella* sp. WBG-1 can readily be used for microalgal mass production coupled with CO_2_ fixation.

## Conclusions

This study identified a lipid-rich microalgal strain *Graesiella* sp. WBG-1. We assessed its growth and lipid production under laboratory and outdoor conditions, and demonstrated that *Graesiella* sp. WBG-1 is an industrial strain capable of high lipid productivity in outdoor mass cultivation. The results can greatly enrich our knowledge of the behavior of oleaginous microalga in outdoor open raceway pond. To the best of our knowledge, this is one of the few reports on integrated indoor and outdoor cultivation for oleaginous microalgae selection. A high lipid content of 33.4 % DW was achieved for the first time in a 200 m^2^ raceway pond. The results also provide evidence for the feasibility of using low-cost raceway pond for autotrophic cultivation of microalgae for lipid production.

## Additional files

10.1186/s13068-016-0541-y Preliminary selection of the 63 microalgae strains in a bubbled column photobioreactor.
10.1186/s13068-016-0541-y Changes of residual nitrate concentration during the experiments.
10.1186/s13068-016-0541-y Daily light irradiance and air temperature during the outdoor trials.
10.1186/s13068-016-0541-y Simulation of biomass-specific light availability in the used reactors based on different volumetric biomass concentration.
10.1186/s13068-016-0541-y Biomass concentration and lipid content of several other microalgae cultured in different PBRs.

## Abbreviations

PBR: photobioreactor
DW: dry weight
*C*_biomass_: dry biomass concentration
*C*_lipid_: lipid content
*P*_biomass_: biomass productivity
*P*_lipid_: lipid productivity
*I*_av_: average light intensity at the PBR surface or culture suspension surface
*I*_biomass_: biomass-specific light availability
*R*_c_: CO_2_ utilization rate
