# Supplementary material for: Effective cultivation of microalgae for biofuel production: a pilot-scale evaluation of a novel oleaginous microalga Graesiella sp. WBG-1
Source: Biotechnol Biofuels. 2016 Jun 13;9:123. doi: 10.1186/s13068-016-0541-y (PMC4906892; doi:10.1186/s13068-016-0541-y)
Supplement: Supplementary file 1 — 10.1186/s13068-016-0541-y Preliminary selection of the 63 microalgae strains in a bubbled column photobioreactor. [file 13068_2016_541_MOESM1_ESM.docx]

Additional file 1: Preliminary selection of the 63 microalgae strains in a bubbled column PBR

| Strain NO. | Lipid productivity | Strain NO. | Lipid productivity |
| --- | --- | --- | --- |
| WBG-1 | 88.5±1.7 | WBG-33 | 24.65±1.3 |
| WBG-2 | 77.2±4.1 | WBG-34 | 65.12±2.8 |
| WBG-3 | 36.02±1.6 | WBG-35 | 28.46±1.2 |
| WBG-4 | 64.85±3.4 | WBG-36 | 44.44±1.9 |
| WBG-5 | 39.91±0.1 | WBG-37 | 24.91±1.3 |
| WBG-6 | 64.39±1.9 | WBG-38 | 38.24±1.2 |
| WBG-7 | 59.02±5.2 | WBG-39 | 11.34±1.2 |
| WBG-8 | 70.11±7.5 | WBG-40 | 55.44±2.6 |
| WBG-9 | 71.56±4.7 | WBG-41 | 50.54±1.6 |
| WBG-10 | 49.37±0.8 | WBG-42 | 46.91±1.9 |
| WBG-11 | 29.43±1.7 | WBG-43 | 49.39±2.6 |
| WBG-12 | 35.14±5.7 | WBG-44 | 29.63±1.6 |
| WBG-13 | 39.47±2.5 | WBG-45 | 42.49±1.7 |
| WBG-14 | 29.6±0.2 | WBG-46 | 39.8±1.1 |
| WBG-15 | 27.59±1 | WBG-47 | 87.54±1.5 |
| WBG-16 | 62.21±0.8 | WBG-48 | 50.18±1.5 |
| WBG-17 | 33.8±0.2 | WBG-49 | 7.76±1.5 |
| WBG-18 | 29.73±1.1 | WBG-50 | 36.84±1.8 |
| WBG-19 | Culture collapsed | WBG-51 | 39.45±1.2 |
| WBG-20 | 4.17±1.2 | WBG-52 | 43.53±1.8 |
| WBG-21 | 39.59±1.3 | WBG-53 | 15.26±2.3 |
| WBG-22 | 34.24±1.1 | WBG-54 | 28.64±1.8 |
| WBG-23 | 28.04±0.8 | WBG-55 | 45.31±1.3 |
| WBG-24 | 30.32±1.9 | WBG-56 | 17.14±2.2 |
| WBG-25 | 29.97±1.1 | WBG-57 | 39.65±1.3 |
| WBG-26 | 33.08±0.5 | WBG-58 | 32.74±1.4 |
| WBG-27 | 26.75±1.2 | WBG-59 | 97.77±2.4 |
| WBG-28 | 31.42±1.7 | WBG-60 | 43.76±1.8 |
| WBG-29 | 56.91±2.2 | WBG-61 | 26.58±1.3 |
| WBG-30 | 28.43±1.2 | WBG-62 | 42.37±1.5 |
| WBG-31 | 34.13±1.6 | WBG-63 | 38.2±1.7 |
| WBG-32 | 45.23±2.7 |  |  |

Note: The PBR used has a working volume of 200 mL (3 cm inner diameter) for each column. During the experiments, the columns were illuminated for 14 hours every day and light intensity at the reactor surface was 300 μmol m^-2^s^-1^. Sterilized air was bubbled with a flow rate of 200 ml min^-1^ for mixing and gas exchange. A thermostatic water circulator was used to provide 30°C water bath for the culture columns. The cultures were grown in batch (8 days) with an initial cell density of 0.5±0.05 (optical density at 540 nm). Initial nitrate concentration was 1.18 mM. Daily average lipid productivities with stand deviation of three replicates are showing in the table.
