# Supplementary material for: Effective cultivation of microalgae for biofuel production: a pilot-scale evaluation of a novel oleaginous microalga Graesiella sp. WBG-1
Source: Biotechnol Biofuels. 2016 Jun 13;9:123. doi: 10.1186/s13068-016-0541-y (PMC4906892; doi:10.1186/s13068-016-0541-y)
Supplement: Supplementary file 2 — 10.1186/s13068-016-0541-y Changes of residual nitrate concentration during the experiments. [file 13068_2016_541_MOESM2_ESM.docx]

Additional file 2: Changes of nitrate concentration during algal cultivation in the 200 mL column PBR (A), 10 L circular pond (B) and 200 m^2^ raceway pond (C)
