# Supplementary material for: Effective cultivation of microalgae for biofuel production: a pilot-scale evaluation of a novel oleaginous microalga Graesiella sp. WBG-1
Source: Biotechnol Biofuels. 2016 Jun 13;9:123. doi: 10.1186/s13068-016-0541-y (PMC4906892; doi:10.1186/s13068-016-0541-y)
Supplement: Supplementary file 3 — 10.1186/s13068-016-0541-y Daily light irradiance and air temperature during the outdoor trials. [file 13068_2016_541_MOESM3_ESM.docx]

Additional file 3: Daily light irradiance and air temperature during the outdoor trials

Solar irradiance (circle) and air temperature (up-triangle) during the outdoor cultivation were logged every 10 min by an automatic weather station. The data from June 12, 2013 to July 15, 2013 were displayed in the figure.
