# Supplementary material for: Effective cultivation of microalgae for biofuel production: a pilot-scale evaluation of a novel oleaginous microalga Graesiella sp. WBG-1
Source: Biotechnol Biofuels. 2016 Jun 13;9:123. doi: 10.1186/s13068-016-0541-y (PMC4906892; doi:10.1186/s13068-016-0541-y)
Supplement: Supplementary file 4 — 10.1186/s13068-016-0541-y Simulation of biomass-specific light availability in the used reactors based on different volumetric biomass concentration. [file 13068_2016_541_MOESM4_ESM.docx]

Additional file 4: Simulation of biomass-specific light availability in the four PBRs used in this study

| Volumetric  biomass  concentration | 200 mL column PBR | | 10 L circular pond | | 30 L tank PBR | | 200 m^2^ raceway pond | |
| --- | --- | --- | --- | --- | --- | --- | --- | --- |
|  | *C_biomass_* | *I_biomass_* | *C_biomass_* | *I_biomass_* | *C_biomass_* | *I_biomass_* | *C_biomass_* | *I_biomass_* |
| 0.5 | 7.9 | 1.91 | 50.0 | 0.30 | 78.9 | 0.39 | 100.0 | 0.40 |
| 0.6 | 9.5 | 1.59 | 60.0 | 0.25 | 94.7 | 0.33 | 120.0 | 0.33 |
| 0.7 | 11.1 | 1.36 | 70.0 | 0.22 | 110.5 | 0.28 | 140.0 | 0.28 |
| 0.8 | 12.7 | 1.19 | 80.0 | 0.19 | 126.3 | 0.25 | 160.0 | 0.25 |
| 0.9 | 14.3 | 1.06 | 90.0 | 0.17 | 142.1 | 0.22 | 180.0 | 0.22 |
| 1.0 | 15.9 | 0.95 | 100.0 | 0.15 | 157.9 | 0.20 | 200.0 | 0.20 |
| 1.5 | 23.8 | 0.64 | 150.0 | 0.10 | 236.8 | 0.13 | 300.0 | 0.13 |
| 2.0 | 31.7 | 0.48 | 200.0 | 0.08 | 315.8 | 0.10 | 400.0 | 0.10 |
| 2.5 | 39.7 | 0.38 | 250.0 | 0.06 | 394.7 | 0.08 | 500.0 | 0.08 |

Note: Areal biomass concentration (*C_biomass_*, g m^-2^) and Biomass-specific light availability (*I_biomass_*, mol g^-1^ DW d^-1^) were calculated supposing the volumetric biomass concentrations (g L^-1^) were 0.5, 0.6, 0.7, 0.8 0.9, 1.0, 1.5, 2.0 and 2.5 g L^-1^ in each reactor. The same light intensities (as in the experiment) of 15.12 mol m^-2^d^-1^ in 200 mL column PBR, 15.12 mol m^-2^d^-1^ in 10 L circular pond, and the historical radiation data of Avg. 31.0 mol m^-2^d^-1^ in 30 L tank PBR, Avg. 39.6 mol m^-2^d^-1^ in 200 m^2^ raceway pond were used in the simulation. It was noted that the calculated *I_biomass_* is the lowest value during the whole culture period.

Biomass-specific light availability (*I_biomass_*) can be calculated by the following equation:

$$I_{biomass}=\frac{I_{av}\text{ }(\mu mol\text{ }m^{-2}s^{-1})}{C_{biomass}\text{ (}g m^{-2}\text{)}}$$

where *I_av_* is the light impinging on the reactor surface, and *C_biomass_* is the biomass concentration per illuminating area. *C_biomass_* can be calculated by:

$$C_{biomass}=\frac{C\text{ }(g\text{ }L^{-1})\times V\text{ }(L)}{S\text{ (m}^{2}\text{)}}$$

where *C* is the volumetric biomass density, *V* and *S* is the culture volume and illuminating area of reactor respectively. For the 200 mL column PBR, *V* and *S* is 200 mL and 0.013m^2^ respectively. For the 10 L circular pond, *V* and *S* is 10 L and 0.1 m^2^ respectively. For the 30 L tank PBR, *V* and *S* is 30 L and 0.19 m^2^ respectively. For the 200 m^2^ raceway pond, *V* and *S* is 40,000 L and 200 m^2^ respectively.
