# Supplementary material for: Effective cultivation of microalgae for biofuel production: a pilot-scale evaluation of a novel oleaginous microalga Graesiella sp. WBG-1
Source: Biotechnol Biofuels. 2016 Jun 13;9:123. doi: 10.1186/s13068-016-0541-y (PMC4906892; doi:10.1186/s13068-016-0541-y)
Supplement: Supplementary file 5 — 10.1186/s13068-016-0541-y Biomass concentration and lipid content of several other microalgae cultured in different PBRs. [file 13068_2016_541_MOESM5_ESM.docx]

Additional file 5: Lipid content of five microalgae strains cultivated in different PBRs. Outdoor cultivation was conducted in summer climate at Chenghai Lake, China. Numbers of the X-axis indicate microalgae strains, which is *Chlorella pyrenoidosa* WBG-A (1), *Scenedesmus obliquus* WBG-B (2), *Chlorococcum* sp. WBG-C (3), *Scenedesmus* sp. WBG-4 (4), *Graesiella* sp. WBG-1 (5), respectively. Dash lines indicated the initial lipid content (nitrogen sufficient) of each microalga.
